# Perspectives on strained intensive care unit capacity: A survey of critical care professionals

**DOI:** 10.1371/journal.pone.0201524

**Published:** 2018-08-22

**Authors:** Dawn Opgenorth, Henry T. Stelfox, Elaine Gilfoyle, R. T. Noel Gibney, Michael Meier, Paul Boucher, David McKinlay, Christiane N. Job McIntosh, Xiaoming Wang, David A. Zygun, Sean M. Bagshaw

**Affiliations:** 1 Department of Critical Care Medicine, Faculty of Medicine and Dentistry, University of Alberta, Edmonton, Alberta, Canada; 2 Department of Critical Care Medicine, Cumming School of Medicine, University of Calgary, Calgary, Alberta, Canada; 3 Critical Care Strategic Clinical Network, Alberta Health Services, Edmonton, Alberta, Canada; 4 Department of Community Health Sciences, Cumming School of Medicine, University of Calgary, Calgary, Alberta, Canada; 5 Section of Critical Care, Department of Pediatrics, Cumming School of Medicine, University of Calgary, Calgary, Alberta, Canada; 6 Research Facilitation, Analytics (DIMR), Alberta Health Services, Edmonton, Alberta, Canada; Azienda Ospedaliero Universitaria Careggi, ITALY

## Abstract

**Background:**

Strained intensive care unit (ICU) capacity represents a supply-demand mismatch in ICU care. Limited data have explored health care worker (HCW) perceptions of strain.

**Methods:**

Cross-sectional survey of HCW across 16 Alberta ICUs. A web-based questionnaire captured data on demographics, strain definition, and sources, impact and strategies for management.

**Results:**

658 HCW responded (33%; 95%CI, 32–36%), of which 452 were nurses (69%), 128 allied health (19%), 45 physicians (7%) and 33 administrators (5%). Participants (agreed/strongly agreed: 94%) reported that strain was best defined as *“a time-varying imbalance between the supply of available beds*, *staff and/or resources and the demand to provide high-quality care for patients who may become or who are critically ill”*; while some recommended defining “high-quality care”, integrating “safety”, and families in the definition. Participants reported significant contributors to strain were: “*inability to discharge ICU patients due to lack of available ward beds”* (97%); “*increases in the volume”* (89%); and “*acuity and complexity of patients requiring ICU support”* (88%). Strain was perceived to “*increase stress levels in health care providers*” (98%); and “*burnout in health care providers*” (96%). The highest ranked strategies were: “*have more consistent and better goals-of-care conversations with patients/families outside of ICU*” (95%); and “*increase non-acute care beds*” (92%).

**Interpretation:**

Strain is perceived as common. HCW believe precipitants represent a mix of patient-related and operational factors. Strain is thought to have negative implications for quality of care, HCW well-being and workplace environment. Most indicated strategies “outside” of ICU settings were priorities for managing strain.

## Introduction

Strained intensive care unit (ICU) capacity represents a demand-supply disparity between availability of critical care resources (i.e., bed availability; bedside resources) and capability to provide high-quality life-sustaining care for patients with critical illness.[1] Numerous studies have suggested that strain exerts a small but clinically important risk for delivery of suboptimal care, altered care processes and adverse outcomes.[2–8]

Prior data have implied that healthcare workers (HCW) believe that strained ICU capacity negatively impacts the workplace environment, predisposes to moral distress and burnout, and contributes to HCW attrition.[9–11] However, few studies have focused on understanding HCW perceptions on strained ICU capacity, in particular related to precipitants, impact on patients, families and HCW, and strategies for prevention and management.[1, 12]

Accordingly, we performed a province-wide survey of inter-professional ICU HCW to describe and explore their beliefs pertaining to strained ICU capacity. This work was performed in a fully integrated single-payer geographically-defined healthcare system serving a population of 4 million residents in Alberta, Canada, where ICUs regularly function at near or full capacity.[13] This survey consolidates our prior qualitative work engaging inter-professional HCW to better understand strained ICU capacity in Alberta with a long-term goal of identifying methods to optimize critical care services delivery and enhance patient care.[1]

## Methods

The study received approval from the Health Research Ethics Board at the University of Alberta (File # Pro00046184). Participation was voluntary and consent implied by completion of the survey.

### Study design, setting and population

We performed a cross-sectional survey targeting inter-professional ICU HCW employed at the 16 mixed medical/surgical ICUs across Alberta, Canada. The survey targeted bedside nurses (i.e. registered nurses [RNs], nurse practitioners [NPs], nurse aides), physicians (MDs), allied health (AH) practitioners (i.e., registered respiratory therapists [RRT], pharmacists, social workers, dieticians, physiotherapists, occupational therapists) and administrators/managers. The survey was conducted between March 21 and May 20, 2016.

### Survey development

Survey content was derived from a prior qualitative study focused on strained ICU capacity involving inter-professional focus groups.[1] Critical care professionals who participated in this qualitative study were not specifically excluded from participating in the survey. The survey was a web-based 70-item questionnaire. The survey integrated socio-demographic factors (6 questions) including age, HCW role, current position (i.e.: full time, part time), specific ICU of practice and years of ICU experience. The survey measured HCW perceptions about strained ICU capacity, including definition (2 questions), contributing factors (18 questions), impact (14 questions) and potential strategies for mitigation and management (30 questions). Survey questions utilized a 5-point Likert scale (1 –*Strongly Disagree* to 5 –*Strongly Agree*) with additional open-ended questions for free text comment. The survey underwent pre-testing, clinical sensibility and pilot testing for clarity, comprehension, redundancy, face validity and administrative ease.[14] The survey was built in Research Electronic Data Capture (REDCap) (Vanderbilt, Tn) to enable a secure web-based electronic link for completion. The link was pilot tested to evaluate connectivity and assess online functionality and flow.

### Survey administration

The survey was distributed to HCW electronically through a central provincial email distribution provided by the Alberta Health Services (AHS) Critical Care Strategic Clinical Network (CCSCN), along with further redundant snowball dissemination through local site-specific leadership. This electronic distribution list was utilized to provide numerator estimates of inter-professional HCW across the 16 ICUs.[15] The CCSCN is an inter-professional committee comprised of physicians, nurses, allied health professionals, managers/decision makers, researchers and patient/family advisors representing all critical care jurisdictions across Alberta (www.criticalcareresearchscn.com).[1] In addition, the survey was promoted through the CCSCN website, through provincial, regional and local newsletters and announcements, and through promotional material (i.e., posters, bedside cards with an electronic link to the survey) disseminated to all participating ICUs. Reminder emails were sent every two weeks during the survey period.

### Analysis

Data analyses were descriptive. No assumptions or imputations were made for missing data. Data were collated and presented as means (SD) and proportions (%). Comparisons of responses across demographic factors, HCW groups, and hospital types (i.e., regional vs urban) were performed using non-paired t-tests and analysis of variance and covariance tests, where applicable. A p-value of <0.05 was considered statistically significant for all comparisons. Simple content analysis of open-ended free text comments was performed to identify common and recurring themes. All analyses were performed using SAS (Release 9.4; SAS Institute, Caryn NC).

## Results

A total of 658 participants completed the survey, for an estimated response rate of 34% (95% CI, 32–36). The most common participants were nurses (n = 452; 69%), followed by AH practitioners (n = 128; 19%), physicians (n = 45; 7%) and administrators (n = 33; 5%) (Table 1). Most participants were aged 26–50 years (n = 507; 77%) and close to half (n = 332; 47%) worked in academic/tertiary hospital ICUs (Table 2). In total, 57% had worked in an ICU for >5 years and 78% had been in their respective ICU for >2 years (Table 2).

**Table 1:** Summary of participant distribution by health care worker type and response rate.

HCW Type	Responses (n, %)	Denominator (n*)	Response Rate (%)
Nurses	452 (69)	1245 (64)	36
Physicians	45 (7)	118 (6)	38
Allied Health	128 (19)	502 (25)	26
Administrators/Managers	33 (5)	91 (5)	37
Total	658 (100)	1956 (100)	34

* estimated number of HCW to be surveyed

**Table 2:** Summary of demographic features of survey participants.

Demographics	n (%)
Age, years	
< 25 yr	56 (8)
26–34 yr	236 (36)
35–50 yr	271 (41)
> 51 yr	95 (15)
ICU Type	
Academic tertiary	332 (47)
Urban community	196 (33)
Rural regional	130 (20)
Years of ICU experience–overall	
<2 years	90 (14)
2–5 years	193 (29)
6–10 years	135 (20)
>10 years	240 (37)
Years of ICU experience–current ICU	
<2 years	146 (22)
2–5 years	195 (30)
6–10 years	144 (22)
>10 years	173 (26)

### Definition of strained ICU capacity

Participants agreed/strongly agreed (n = 608; 94%) that strained ICU capacity was best conceptually defined as *“a time-varying imbalance between the supply of available beds*, *staff and/or resources and the demand to provide high-quality care for patients who may become or who are critically ill”*. In content analysis of open text comments, participants suggested additional aspects be considered in a definition of strained ICU capacity. These included greater clarity to defining “high-quality care” along with integrating the concept of “safety” (participant quote *“I would include “safe” to the demand [side of strained ICU capacity] to provide needed high-quality care”*). Participants further suggested the definition should explicitly integrate care for not only patients but also their families.

### Contributors to strained ICU capacity

**Fig 1:**
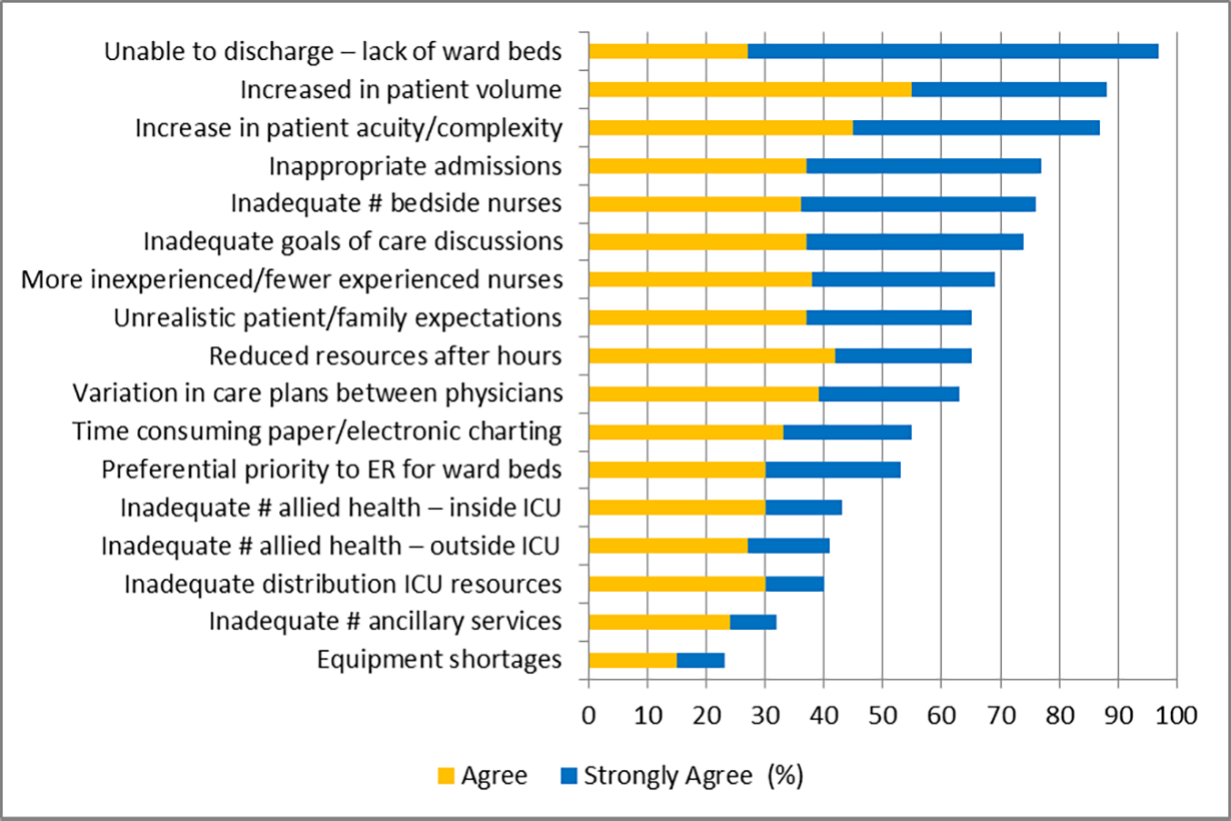
Summary of participant perceptions of contributors to strained ICU capacity.

More than 50% of respondents agreed/strongly agreed that 12 (71%) of the precipitants identified in prior focus groups contributed to strained ICU capacity (Fig 1).[1] Participants reported the most significant contributors to be: *inability to discharge ICU patients due to lack of available ward beds* (97%); *increases in the volume of patients requiring ICU support* (89%); *increases in the acuity and complexity of patients requiring ICU support* (88%); and *insufficient bedside nursing coverage to manage workload* (79%). The least important contributors identified by participants were: *under-utilization of regional ICU resources* (40%); *unavailability of ancillary service personnel* (i.e., housekeeping; porters) (32%); and *equipment shortages* (i.e., ventilators) (23%).

In analysis of individual HCW group responses, all groups ranked “*inability to discharge ICU patients due to lack of available ward beds*” as the greatest perceived contributor to strained ICU capacity. The second highest ranked contributors to strained ICU capacity across HCW were for nurses “*inadequate number of bedside nurses to cover the patient care workload*” (agree/strongly agree: 87%; n = 425); for AH and administrators, both ranked “*an increase in the overall volume of patients requiring ICU-level care*” (AH—agree/strongly agree: 87%; n = 120; administrators—90%; n = 31); and for physicians “*an increase in the acuity and complexity of patients admitted to the ICU*” (agree/strongly agree: 86%; n = 44). All HCW groups ranked “*equipment shortages*” and “*inadequate ancillary service personnel*” as the lowest perceived contributors to strain.

In analysis of open text comments, participants further suggested that the timeliness of multi-disciplinary daily patient rounds and staff breaks can interrupt work flow and contribute to delays in patient readiness for ICU discharge/ward transfer (participant quote *“[the ICU often experiences] inherent workflow inefficiency”* and “*variation in the [daily multi-disciplinary team rounding] can delay [development of care] plans for discharge and [timely transition for ward ready patients]*”).

### Impact of strained ICU capacity

**Fig 2:**
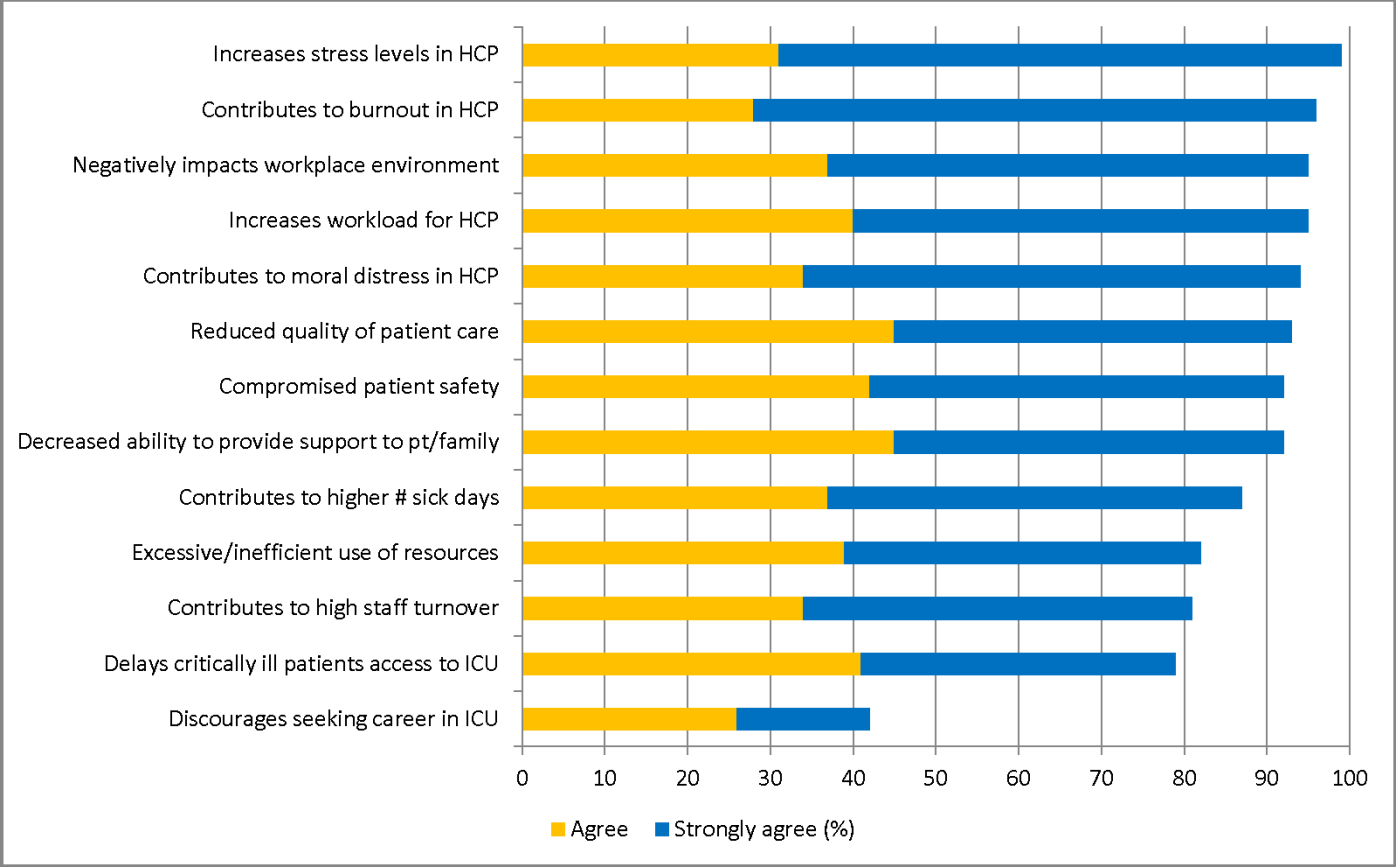
Summary of participant perceptions of the impact of strained ICU capacity.

More than 50% of participants agreed/strongly agreed that 12 (92%) of the proposed items were significantly impacted by strained ICU capacity (Fig 2). Strain was perceived to impact “*increased stress levels in health care providers*” (agree/strongly agree: 98%; n = 623); contribute to “*burnout in health care providers*” (agree/strongly agree: 96%; n = 621); and “*negatively impacts the workplace environment*” (agree/strongly agree: 95%; n = 621). This was associated with 42% (n = 623) of participants believing strain “*discourage[d] health care workers from seeking a career in ICU*”. This was significantly higher for nurses (46%; n = 425) compared with physicians (31%; n = 45; p = 0.012). Notably, HCW perceived strain compromised quality of patient care (agree/strongly agree: 93%; n = 597) and patient safety (agree/strongly agree: 92%; n = 572). This was significantly greater for nurses when compared to physicians (p<0.001 for each), to AH providers (p<0.001 for each) and to administrators (p = 0.03; p<0.001), respectively.

All HCW groups consistently perceived that strain “*increased stress levels in health care providers”*. This was highest ranked for AH (agree/strongly agree: 96%; n = 120); administrators (agree/strongly agree: 97%; n = 31) and physicians (agree/strongly agree: 93%; n = 44). In addition to increased stress levels, nurses strongly perceived strained ICU capacity “*contributes to burnout in health care providers*” (agree/strongly agree: 98%; n = 425) and “*contributes to moral distress in health care providers*” (agree/strongly agree: 96%; n = 425). All HCW groups, who notably already work in the ICU, ranked “*discourages seeking a career in the ICU*” as the lowest ranked impact of strain.

Content analysis of open text comments suggested additional themes focused on the impact of strained ICU capacity. Participants suggested that both patients and families can perceive when ICUs are strained and that this contributes to unnecessary and intensified stress, anxiety and dissatisfaction (participant quotes: “*patients [and families] get frustrated also*, *as they feel…more one on one care and attention [is needed]*”; “*[strained ICU capacity] increases patient and family stress and anxiety*. *An overly busy ICU is intimidating*”; and “*families feel neglected and become resentful towards overworked nurses*”). Participants also commented that strained ICU capacity can contribute to “*physician disengagement*” and may “*negatively impact [health care provider’s] families*”.

### Strategies to mitigate and manage strained ICU capacity

**Fig 3:**
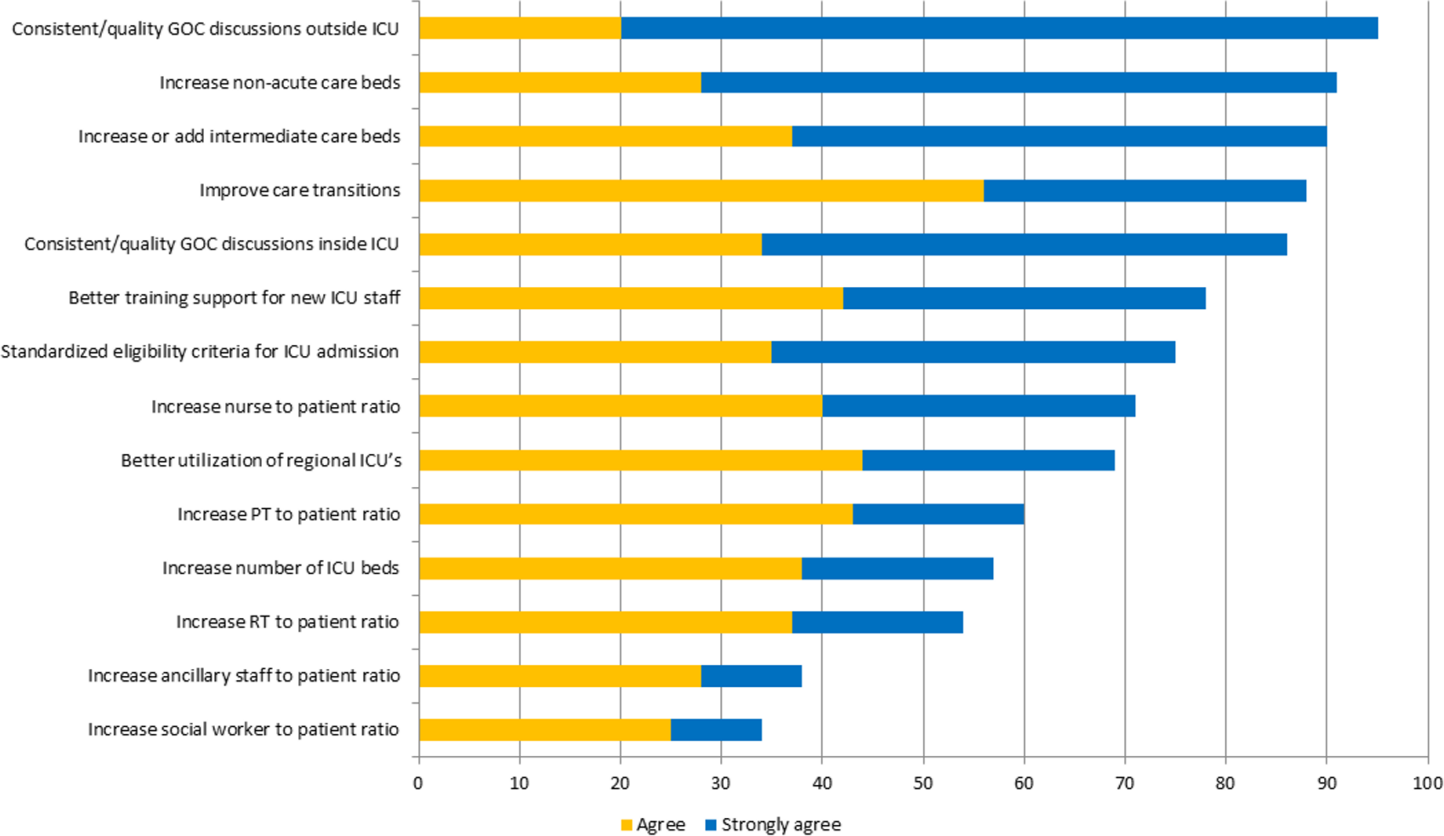
Summary of participant perceptions of strategies to mitigate and manage strained ICU capacity.

**Fig 4:**
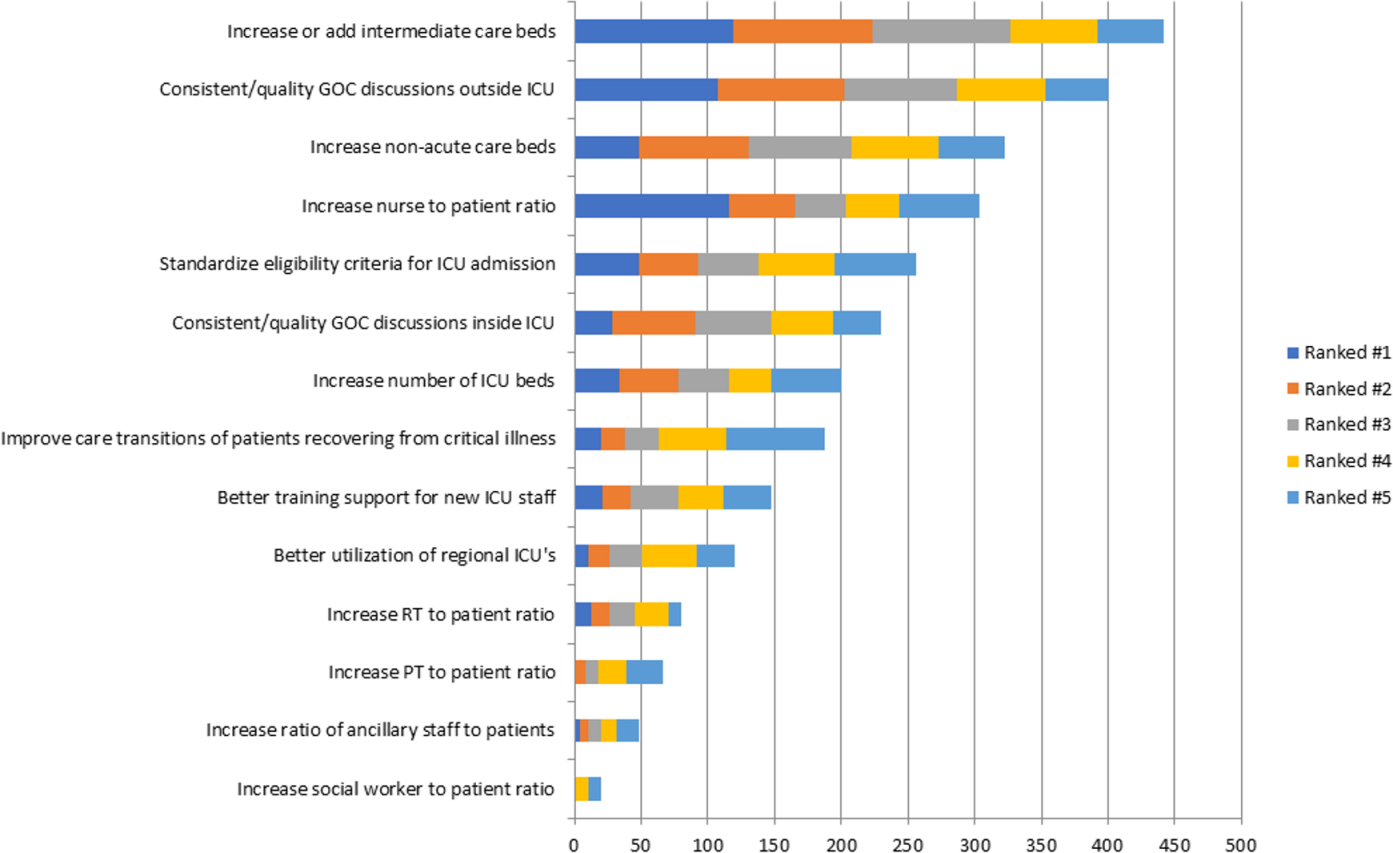
Ranking of participant perceptions of the most effective strategies to potentially mitigate and manage strained ICU capacity.

More than 50% of participants agreed/strongly agreed with 12 (86%) of the proposals to mitigate and manage strain (Figs 3 and 4). The highest ranked strategies across all HCW groups were: “*have more consistent and better goals-of-care conversations with patients and families outside of the ICU*” (agreed/strongly agreed: 95%; n = 615); “*increase the number of non-acute care beds (i*.*e*., *long-term care*, *mental health*, *assisted living)*” (agreed/strongly agreed: 92%; n = 612); and “*add or increase the number of step-down or intermediate care beds*” (agreed/strongly agreed: 91%; n = 611). The lowest ranked strategies were “*increase the ratio of social workers to patients*” (disagreed/strongly disagreed: 28%; n = 617) and “*increase the ratio of ancillary staff to patients (i*.*e*., *service aids*, *housekeeping*, *porters)*” (disagreed/strongly disagreed: 27%; n = 598). While these were generally ranked low across al HCW groups, both strategies were ranked significantly higher for nurses, AH and administrators when compared to physicians (p<0.001). All HCW groups consistently ranked “*have more consistent and better goals-of-care conversations with patients and families outside of the ICU*” as the highest perceived strategy to potentially manage strained ICU capacity.

**Fig 5:**
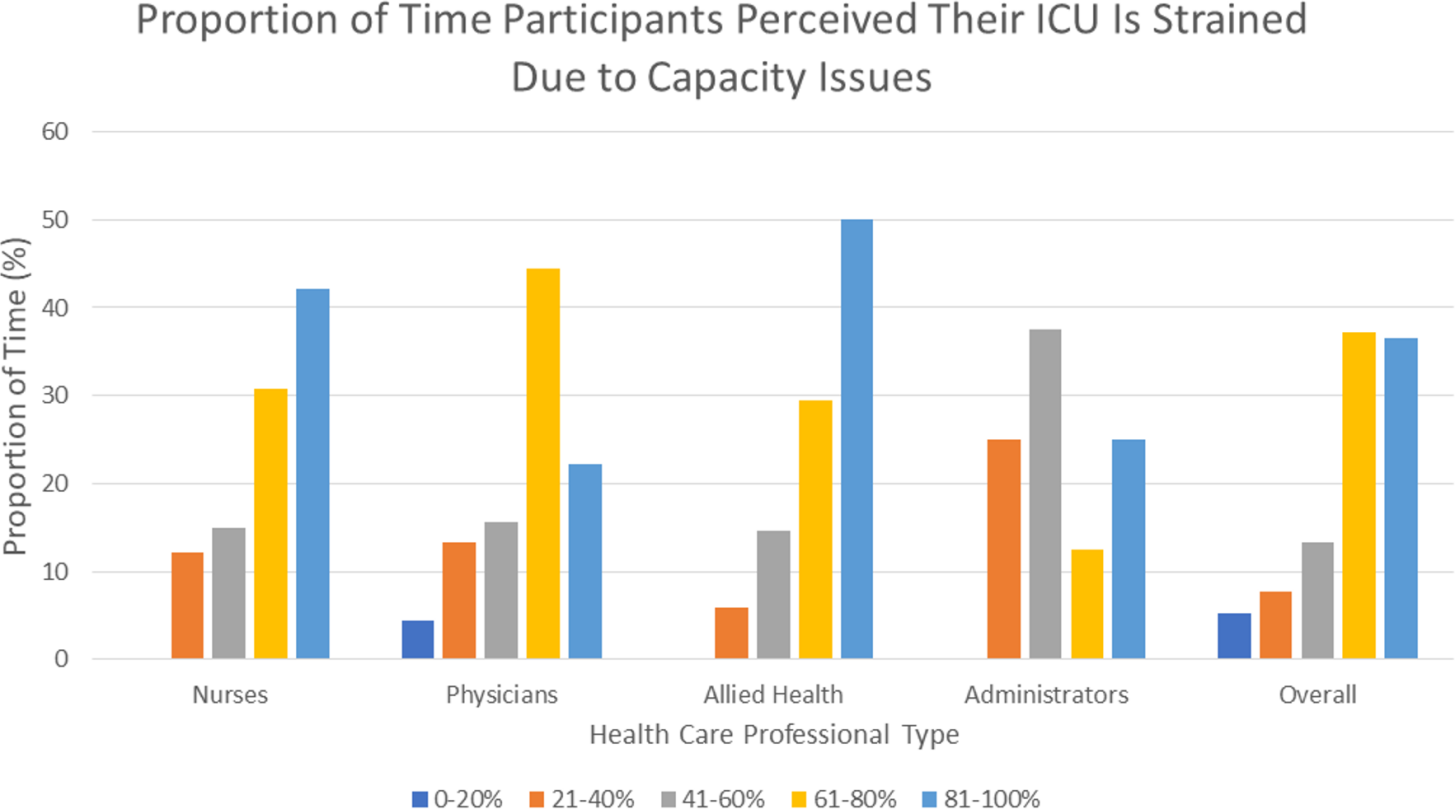
Summary of the proportion of time health care workers perceived their ICU is strained due to capacity issues.

When asked, 37% of participants estimated their ICU experienced strained capacity 80–100% of the time (Fig 5). AH providers perceived the greatest strain (ICU strained 80–100%: 50%; n = 115), whereas administrators perceived the lowest strain (ICU strained 80–100%: 25%; n = 31).

## Discussion

In our provincial survey, we aimed to expand our knowledge of inter-professional ICU healthcare worker’s perceptions of strained ICU capacity. Active engagement of bedside ICU stakeholders is fundamental to appreciate their unique perspectives on the performance of the health system, to recognize opportunities for improving patient care, and gain insight into sustainable and workable strategies to manage strained ICU capacity.

### Key findings

Most participants agreed that strained ICU capacity represents a fundamental mismatch in the supply-demand relationship for patients with critical illness and the capability to provide high-quality and safe care. Participants indicated that any definition for strain should also define the scope and quality of ICU care and explicitly acknowledge the role of patients’ families. We found the greatest perceived factors contributing to strain focused on aspects of operations management (i.e., increased patient admissions; patient flow through the ICU; bedside staffing) and patient-related factors (i.e., multi-morbidity; high acuity; treatment complexity). We found all HCW groups believed that strained ICU capacity contributes to excess HCW stress, predisposes to burnout and has negative implications for workplace environment. Moreover, HCW, particularly nurses strongly believed strain compromised the quality and safety of patient care. We found that 2 out of every 5 respondents believed that strained ICU capacity discouraged others from seeking a career in ICU, more so for nurses. Participants also believed that both patients and families recognized when ICUs were strained and that it contributed to their perception of receiving lower quality of care and their dissatisfaction. Proactive engagement in goals-of-care discussions with patients and families prior to critical illness and ICU admission were consistently perceived as a strategy likely to help mitigate strain. While ICU settings are generally acknowledged as relatively high-stress time-pressured environments, a significant proportion believed their respective ICUs operated under excessively strained conditions a large majority of the time.

### Context with prior studies

Reduced patient flow through the ICU, particularly “bed-block” or medically unnecessary ICU discharge delays due to lack of available ward beds was strongly perceived by HCW as an important mechanism triggering strain. We submit this indicator of strain can be readily measured at the ICU level as “avoidable-days”, defined as the proportion of total ICU stay (and cumulative patient-days) accounted for by avoidable delay in ICU discharge.[16, 17] Avoidable delays in ICU discharge may have negative implications for care processes and outcomes for patients.[18] Avoidable-days, as a key performance indicator, can be used to identify operational inefficiencies and target quality improvement initiatives. HCW also reported provider-related factors played a role in ICU transfer delays and flow failure, such as variability in ICU rounding practice.[19] Interruptions and prolonged rounding time may be important barriers to efficient daily rounding practice.[20] Though, daily rounds may be negatively impacted by strained capacity itself, particularly when ICUs are characterized by high occupancy, new admissions or patients with high illness acuity.[7] Non-patient-related factors, such as operational inefficiencies, remain a common cause for flow failure and transfer delays, which have been associated with unnecessary hazard to patients.[18, 21]

Temporal changes in patient-related factors were also strongly perceived as contributors to strained ICU capacity. These “demand-side” influences, such as growing requests for ICU support or greater patient multi-morbidity, acuity and complexity correlate directly with “supply-side” issues, particularly bedside workload. Nurses strongly perceived strain intensifies their workload, and negatively impacts quality and safety of patient care. Excessive bedside workload has been associated with reduced quality of care and increased risk for patient morbidity.[2] While determinants of excessive workload are complex, strained conditions, particularly in context of nursing inexperienced and/or insufficient bedside staffing, synergistically may amplify the perception of HCW workplace stress.[22, 23] The perception that ICUs are persistently strained, as implied by participants, was also implicated in adversely affecting the workplace environment and risk of burnout.[9, 10] Collectively, these factors may propagate a negatively reinforcing feedback cycle of strain, burnout and attrition among HCW.

Participants also believed that patients and families recognize when ICUs are strained; however, no prior work has specifically evaluated the impact of strain on patient and family satisfaction with their ICU care and experience. Prior data have suggested families have generally been very satisfied with the emotional support, compassion and bedside care provided by nurses for both patients and families; while least satisfied by physician communication and the “waiting room” environment.[24–26] Strain could plausibly worsen family satisfaction, both with ICU care in general and in their perception of being engaged as a member of the ICU team. These notions are reinforced by suggestion that any conceptual definition of strained ICU capacity should integrate care both patient and their families.

Of strategies offered to HCW, those perceived to have the greatest impact to mitigate and/or manage strain were largely focused outside of ICU settings. ICU HCW strongly believed that patients and families should better understand the implications of ICU care and have had better opportunity for informed goals-of-care discussions prior to development of critical illness or clinical deterioration. These observations would suggest that inter-professional HCW strongly perceive strained ICU capacity may be in part attributed to the quality and timeliness of communication between patients and/or families and their health care teams. Prior data have shown communication about end-of-life care preferences are often suboptimal.[27] Barriers to effective engagement in advance care planning (ACP) and goals-of-care discussion are largely perceived by HCW to be patient and/or family member-related.[28] However, ACP has been shown to reduce undesired intensification of care at end-of-life and improve patient/family satisfaction with care.[29] These are likely opportunity costs and could reduce unnecessary demand. Alternatively, the suggestion to expand intermediate care or non-acute care beds aligns directly with HCW belief that bed-block and “avoidable-days” was a common and critical precipitant to supply-side strain. While these may represent important long-term approaches to mitigate strain, ICUs must also consider development of short-term strategies, including recognizing and acknowledging strain and managing the resultant HCW stress.

### Implications for research

Strained ICU capacity has generally been indirectly implied through routine measures of ICU performance (i.e., occupancy); however, the potential subtle and varied effects of strain across the whole of the ICU environment has been less rigorously interrogated. Recently, validated measures of “staff work life” and “family satisfaction” have been recommended for integration as key indicators of ICU performance.[30] This would enable future work to correlate objective periods of strain with measures of HCW well-being and staff turnover.[10, 31] Similarly, we submit future research should aim to learn whether and how patients/families perceive busy ICUs and to correlate operational indicators of strain with validated measures of family satisfaction.

### Strengths and limitations

Our study has both strengths and limitations. Our survey was rigorously developed, including the integration of content derived from inter-professional focus groups, along with undergoing pilot and clinical sensibility testing.[1] Our survey also targeted a broad range of inter-professional ICU HCW and was provincial in scope. Despite these strengths, we had to estimate the provincial sampling frame (i.e., denominator) and from this our response rate was suboptimal, though comparable to other population-based HCW surveys.[32] Second, we utilized snowball sampling as a secondary strategy to target participants. As such, our results are susceptible to sampling bias, possibly related to both those who did and those who did not respond. For example, those responding may have been more engaged and willing to express opinions about strained ICU capacity. Alternatively, those who did not respond may have also been concerned about strained ICU capacity; however, unable or unwilling to respond due to limited opportunity to complete the survey. Third, while our survey underwent pre-testing, clinical sensibility testing and piloting, we recognize our survey was novel and not a validated instrument. Finally, we presented HCW with a conceptual definition for strained ICU capacity derived from prior focus group content. We then solicited their level of agreement with the proposed definition and whether additions, modifications or omissions should be considered. We recognize this may have limited respondent’s opportunity to prioritize aspects of the conceptual definition as presented.

## Conclusion

Strained capacity is perceived as common among inter-professional ICU HCW. Most suggest the precipitants of strain represent a mixture of patient-related and operations-related factors (i.e., patient flow). Strain is strongly believed to have negative implications on quality of care, on HCW well-being and on the ICU workplace environment. Strategies focused outside of direct ICU settings were perceived as key priorities for managing strain. These findings should help prioritize and direct initiatives aimed at managing strained capacity in ICUs across Alberta.

## Supporting information

S1 File Questionnaire tool. (PDF)
