# Supplementary material for: Perspectives on strained intensive care unit capacity: A survey of critical care professionals
Source: PLoS One. 2018 Aug 22;13(8):e0201524. doi: 10.1371/journal.pone.0201524 (PMC6104911; doi:10.1371/journal.pone.0201524)
Supplement: S1 File — (PDF) [file pone.0201524.s001.pdf]

IDENTIFYING AND EVALUATING THE IMPACT OF INTENSIVE CARE UNIT CAPACITY STRAIN

<u>HEALTH CARE PROVIDER SURVEY</u>

Title: Identifying and evaluating the impact of Intensive Care Unit (ICU) capacity strain

Principal Investigators: Dr. Sean M Bagshaw, Dr. H. Tom Stelfox

Dear Colleague,

We invite you to participate in this brief survey focused on identifying the causes and impact of capacity strain in the ICU.

Capacity strain occurs when the demand for access to ICU services is high but the supply is fixed and limited. This mismatch in supply and demand creates "strain" on our health care system.

We want to learn more from health care providers about their experiences, perceptions and beliefs regarding strain on ICU capacity. The information you provide in this survey will help us in our goal to devise strategies to manage capacity strain and ultimately improve care of ICU patients.

- The survey will take approximately 10 minutes to complete.
- Your participation is voluntary and completion of the survey implies consent to participate.
- There are no direct benefits and/or risks to your participation.
- Your responses will be anonymous, kept confidential and only be reported in group summaries. Individual responses will not be reported. The final survey data will be stored by the Principle Investigator (Dr. Bagshaw) on a password protected computer for 5-years.

Survey respondents can choose to have their name entered into a **weekly draw for $20 Starbucks gift cards**. Non-winners will remain in the draw for the following week. The odds of winning a gift card are approximately 1 in 40. To maintain your confidentiality, the contact information you provide for the draw will be separated from your survey responses and deleted after all draws are made.

We appreciate your time and important contribution. The success of this project depends on the generous help of health care providers like you. If you have any questions or concerns please do not hesitate to contact a member of our study team:

Sean M Bagshaw
Principal Investigator
bagshaw@ualberta.ca

Dawn Opgenorth
Project Manager
dawno@ualberta.ca

The Health Research Ethics Board (HREB) at the University of Alberta in Edmonton and the Conjoint Health Research Ethics Board at the University of Calgary (CHREB) have reviewed this study. If you have questions about your rights as a participant, you may contact the HREB at (780) 492-2615 or CHREB at (403) 220-7990. These offices have no affiliation with the study team.

DEMOGRAPHICS

1. What is your role in the ICU?

◯ ICU physician	◯ Respiratory Therapist	◯ Administrator
◯ Registered Nurse	◯ Physiotherapist	◯ Pharmacist
◯ Nurse Practitioner	◯ Social Worker	◯ Other:________________________

2. How would you describe your current position?

☐ Full time
☐ Part time

3. In which hospital do you primarily work?

◯ Chinook Regional Hospital
◯ Foothills Medical Centre
◯ Grand Praire QE II Hospital
◯ Grey Nuns Hospital
◯ Medicine Hat Regional Hospital
◯ Misericordia Hospital
◯ Northern Lights Regional Health Centre
◯ Peter Lougheed Centre
◯ Red Deer Regional Hospital
◯ Rockyview General Hospital
◯ Royal Alexandra Hospital
◯ South Health Campus
◯ Sturgeon Community Hospital
◯ University of Alberta Hospital

4. Overall, how long have you worked in Critical Care?

◯ Less than 2 years
◯ 2-5 years
◯ 6-10 years
◯ More than 10 years

5. **How long have you worked in your current ICU?**
   - ◯ Less than 2 years
   - ◯ 2-5 years
   - ◯ 6-10 years
   - ◯ More than 10 years

6. **What is your age category?**
   - ◯ 25 years or younger
   - ◯ 26 - 34 years
   - ◯ 35 - 50 years
   - ◯ 51 – 65 years
   - ◯ over 65 years

DEFINITION OF CAPACITY STRAIN

7. **Please indicate your level of agreement with the following statement:**

	Strongly Disagree	Disagree	Undecided	Agree	Strongly Agree
Capacity strain is defined as a time-varying imbalance between supply of available beds, staff and/or resources and the demand to provide needed high-quality care for patients who may become or who are critically ill.	◯	◯	◯	◯	◯

Do you believe additional elements should be considered in the definition of Capacity Strain? If so please specify:

CAUSES OF CAPACITY STRAIN

THE FOLLOWING HAVE BEEN IDENTIFIED AS POTENTIAL CONTRIBUTING FACTORS TO CAPACITY STRAIN. INDICATE YOUR LEVEL OF AGREEMENT FOR EACH EXAMPLE AS IT RELATES TO YOUR ICU.

		Strongly Disagree	Disagree	Undecided	Agree	Strongly Agree
8	An increase in the overall volume of patients requiring ICU-level care.	○	○	○	○	○
9	An increase in the acuity and complexity of patients admitted to the ICU.	○	○	○	○	○
10	Inappropriate admissions to the ICU (i.e. patients who are unlikely to benefit from ICU-level support).	○	○	○	○	○
11	Inability to discharge patients to the ward due to lack of available beds.	○	○	○	○	○
12	Inadequate number of ancillary service personnel such as cleaning staff and porters.	○	○	○	○	○
13	Inadequate number of allied health professionals. (i.e. physiotherapists, respiratory therapists, social workers etc.) INSIDE the ICU.	○	○	○	○	○
14	Inadequate number of allied health professionals. (i.e. physiotherapists, respiratory therapists, social workers etc.) OUTSIDE the ICU.	○	○	○	○	○
15	Inadequate number of bedside nurses to cover patient care workload (i.e. double assignments, coverage for coffee/meal breaks).	○	○	○	○	○
16	Increased numbers of less experienced ICU nurses and a shortage of more experienced nurses to mentor them.	○	○	○	○	○
17	Reduced resources after hours at night and on weekends/holidays (i.e. physiotherapy, respiratory therapy, diagnostic imaging, etc.)	○	○	○	○	○
18	Patients' or families' unrealistic expectations about the scope of care that the ICU can reasonably provide.	○	○	○	○	○
19	Inadequate discussion with patients and/or families regarding goals and preferences for care.	○	○	○	○	○

Causes of Capacity Strain (continued)

INDICATE YOUR LEVEL OF AGREEMENT FOR EACH EXAMPLE AS IT RELATES TO YOUR ICU

		Strongly Disagree	Disagree	Undecided	Agree	Strongly Agree
20	Variation in day-to-day or week-to-week care plans for patients between physicians.	◯	◯	◯	◯	◯
21	Inadequate distribution and utilization of critical care resources (i.e. under-utilization of ICU's in regional centers).	◯	◯	◯	◯	◯
22	Preferential priority given to emergency department when assigning ward beds.	◯	◯	◯	◯	◯
23	Time-consuming patient care documentation (i.e. paper or electronic medical records).	◯	◯	◯	◯	◯
24	Equipment shortages (i.e. ventilators)	◯	◯	◯	◯	◯

25. If you think there are contributors to ICU Capacity Strain that haven't been mentioned above, please list them here:

IMPACT OF CAPACITY STRAIN

THE FOLLOWING ARE EXAMPLES OF POTENTIAL IMPLICATIONS AND IMPACTS OF CAPACITY STRAIN. INDICATE YOUR LEVEL OF AGREEMENT FOR EACH EXAMPLE AS IT RELATES TO YOUR ICU.

**Strain on Capacity…**		Strongly Disagree	Disagree	Undecided	Agree	Strongly Agree
26	Reduces the quality of patient care.	○	○	○	○	○
27	Compromises patients' safety and puts them at higher risk of adverse events.	○	○	○	○	○
28	Reduces health care providers' ability to provide support to patients and their families.	○	○	○	○	○
29	Delays critically ill patients' access to ICU care.	○	○	○	○	○
30	Causes a large or increased workload for health care providers.	○	○	○	○	○
31	Causes increased stress levels in health care providers.	○	○	○	○	○
32	Contributes to moral distress in health care providers.	○	○	○	○	○
33	Contributes to burnout in health care providers.	○	○	○	○	○
34	Negatively impacts workplace environment.	○	○	○	○	○
35	Contributes to high staff turnover.	○	○	○	○	○
36	Contributes to higher numbers of staff sick days.	○	○	○	○	○
37	Discourages health care workers from seeking a career in the ICU.	○	○	○	○	○
38	Causes excessive and inefficient use of resources in the health care system.	○	○	○	○	○

39. If you think there are impacts of ICU Capacity Strain that haven't been mentioned above, please list them here:

SOLUTIONS TO CAPACITY STRAIN

THE FOLLOWING ARE POSSIBLE STRATEGIES TO PREVENT AND/OR REDUCE CAPACITY STRAIN. INDICATE YOUR LEVEL OF AGREEMENT FOR EACH EXAMPLE AS IT RELATES TO YOUR ICU.

		Strongly Disagree	Disagree	Undecided	Agree	Strongly Agree
40	Increase the ratio of bedside nurses to patients.	◯	◯	◯	◯	◯
41	Increase the ratio of respiratory therapists to patients.	◯	◯	◯	◯	◯
42	Increase the ratio of physiotherapists to patients.	◯	◯	◯	◯	◯
43	Increase the ratio of social workers to patients.	◯	◯	◯	◯	◯
44	Increase the ratio of ancillary staff to patients (i.e. service aids, housekeeping, porters)	◯	◯	◯	◯	◯
45	Increase the number of ICU beds.	◯	◯	◯	◯	◯
46	Add or increase the number of step-down or intermediate care beds.	◯	◯	◯	◯	◯
47	Increase the number of non-acute care beds (long term care, mental health, assisted living etc.)	◯	◯	◯	◯	◯
48	Have more consistent and better quality Goals of Care conversations with patients and families INSIDE the ICU.	◯	◯	◯	◯	◯
49	Have more consistent and better quality Goals of Care conversations with patients and families OUTSIDE the ICU.	◯	◯	◯	◯	◯
50	Improve or increase training and support for new ICU staff.	◯	◯	◯	◯	◯
51	Better utilization of regional ICU's	◯	◯	◯	◯	◯
52	Develop standardized eligibility criteria for ICU admission	◯	◯	◯	◯	◯
53	Improve care transitions of patients recovering from critical illness.	◯	◯	◯	◯	◯

54. If you think there are strategies to prevent or reduce ICU Capacity Strain that haven't been mentioned above, please list them here:

SOLUTIONS TO CAPACITY STRAIN – RANKING

BELOW IS A LIST OF POSSIBLE STRATEGIES TO REDUCE OR MANAGE CAPACITY STRAIN. RANK THE FIVE THAT YOU THINK WOULD BE MOST EFFECTIVE.						
		Priority one (highest)	Priority two	Priority three	Priority four	Priority five
55	Increase the ratio of bedside nurses to patients.					
56	Increase the ratio of respiratory therapists to patients.					
57	Increase the ratio of physiotherapists to patients.					
58	Increase the ratio of social workers to patients.					
59	Increase the ratio of ancillary staff to patients (i.e. service aids, housekeeping, porters)					
60	Increase the number of ICU beds.					
61	Add or increase the number of step-down or intermediate care beds.					
62	Increase the number of non-acute care beds.					
63	Improve Goals of Care conversations INSIDE the ICU.					
64	Improve Goals of Care conversations OUTSIDE the ICU.					
65	Improve training and support for new ICU staff					
66	Better utilization of regional ICU's					
67	Develop standardized eligibility criteria for ICU admission					
68	Improve care transitions of patients recovering from critical illness.					

69. If there are strategies that you would rank higher than those listed above, please list them here:

70. In your opinion what percentage of time is your ICU strained due to capacity issues?
______%

If you would like to enter a weekly draw for $20 Starbucks coffee cards please provide your name and contact information (email address or phone #).

Name:
Contact info:

Your contact information will not be linked to your survey answers and will be deleted once all cards are drawn.

Send completed surveys through AHS mail in a <u>sealed</u> envelope to:
Dawn Opgenorth, Critical Care Research
University of Alberta Hospital
2-124, Clinical Sciences Building
8440, 112 Street   Edmonton, AB
T6G 3B7
